# CRISPR, chimerism, and chromothripsis: A technique for studying DNA repair in plants

**DOI:** 10.1093/plcell/koad221

**Published:** 2023-08-21

**Authors:** Andrew C Willoughby

**Affiliations:** Assistant Features Editor, The Plant Cell, American Society of Plant Biologists; Department of Biology, University of North Carolina at Chapel Hill, Chapel Hill, NC 27713, USA

The discovery of Cas9 nuclease has revolutionized the field of gene editing, allowing for precise and programmable modifications to DNA. Many gene-editing techniques stemming from CRISPR-Cas9 (CRISPR) rely on the creation of double-stranded breaks (DSBs) in DNA. However, DSBs pose a threat to the replication process and therefore need to be repaired. The repair of CRISPR-induced DSBs can have varying effects on the genome, with nonhomologous end joining causing small insertions or deletions at the site of the break and homologous recombination potentially leading to crossovers between homologous chromosomes or gene conversion. Targeted crossovers and gene conversion are highly sought-after outcomes in plant breeding for segregating undesirable alleles. These events can enable targeted gene replacement or the elimination of undesirable transgenes, making this process invaluable to the improvement of crops (He et al. 2022). **Aviva Samach, Fabrizio Mafessoni, Or Gross, and colleagues (Samach et al. 2023)** present an assay that detects loss of heterozygosity (LOH) and can be used to study the genomic consequences of DSBs. They induce and can visually identify targeted crossover induction (see Fig.) and also the occurrence of chromothripsis, a process not well studied in plants but characterized by large and severe chromosome rearrangements. This visual assay adds new uses to a growing group of unconventional tools that reduce the need for microscopes or other equipment for particular experiments and screens (Liu et al. 2019, Chen et al. 2023, Sun et al. 2023). These may lower financial and infrastructure barriers to plant biology research, expanding the abilities of universities, field sites, and classrooms.

**Figure.**
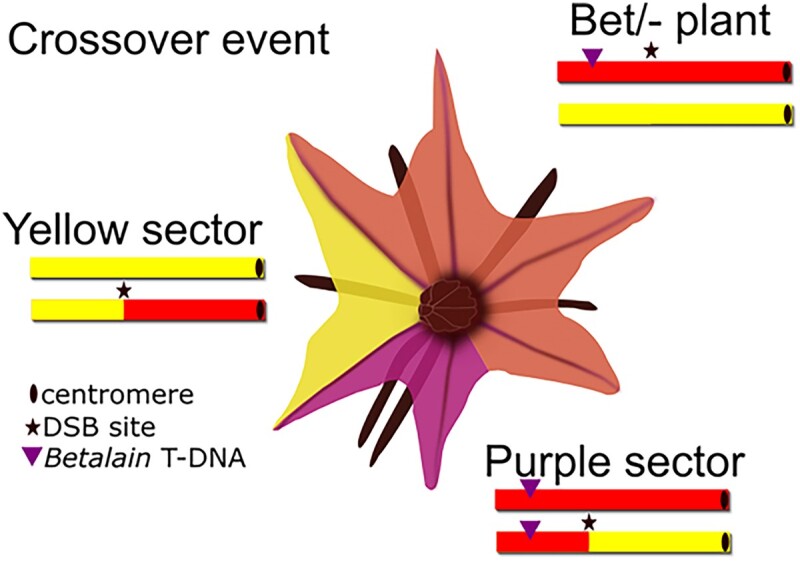
Loss of heterozygosity can be detected by a visual *Betalain* marker inserted into a mapped location. Following induction of DNA breaks by CRISPR, if a crossover occurs between homologous chromosomes, then twin yellow/purple sectors will form distinguished by the copy number of the *Betalain* marker. Adapted from Samach et al. (2023), Figure 3.

The authors used tomato plants with a visible reporter that synthesizes the purple pigment betalain (*Bet*) and mapped the hemizygous insertion of that construct in the genome. They designed guides for targeted DSB break induction by CRISPR between the *Bet* marker and the centromere. If a DSB occurs at that location and results in a crossover, then the resulting LOH can be observed as a twin sector, namely a segment of intensively pigmented *Bet/Bet* tissue adjacent to a segment of −/− pigment-free tissue (see Fig). The loss of the *Betalain* marker through somatic crossover enables the selection of transgene-free plants that contain the desired recombination.

Screening hemizygous light-purple Bet/− plants resulted in the identification of a flower on one plant with a twin sector of dark-purple *Bet/Bet* paired with a yellow −/− sector (see Fig). Because the large sectors likely happened early during flower development, the potential crossover event could be passed down by seed. The authors sequenced progeny from this flower and identified a targeted crossover event at the location of the DSB site, demonstrating transmission of this somatic event.

Due to the rarity of identifying crossovers by sectors, the authors additionally tissue-cultured *Bet/−* seedlings to screen for green plantlets resulting from LOH. After excluding green plants with transgene silencing, they isolated plants with LOH resulting from CRISPR-induced DSBs. They identified a broad range of CRISPR-induced DSB consequences, from the loss of chromosomal fragments to whole chromosome loss. Additionally, they demonstrate that, like in mammals, CRISPR-induced DNA breaks can cause chromothripsis in plants, seen as micronuclei, translocations, dicentric chromosomes, and McClintock's breakage–fusion–bridge cycle. These results and the visual/phenotype-based LOH assay improve our understanding of the genetic and epigenetic context that determines the fate of CRISPR-induced DSBs. This will contribute to more effective use of genome editing in plant breeding applications.

## Dive Curated Terms

The following phenotypic, genotypic, and functional terms are of significance to the work described in this paper:

Betalain CHEBI: CHEBI:3079
